# Validation of the French version of the LEIPAD in community-dwelling people aged 80 years and above

**DOI:** 10.1371/journal.pone.0213907

**Published:** 2019-03-19

**Authors:** Isabelle Jalenques, Candy Guiguet-Auclair, Laurent Gerbaud, Chloé Rachez, Fabien Rondepierre

**Affiliations:** 1 Université Clermont Auvergne, CHU Clermont-Ferrand, Service de Psychiatrie de l’Adulte A et Psychologie Médicale, Centre Mémoire de Ressources et de Recherche, Clermont-Ferrand, France; 2 CHU Clermont-Ferrand, Service de Santé Publique, Clermont-Ferrand, France; 3 CHU Clermont-Ferrand, Service de Psychiatrie de l’Adulte A et Psychologie Médicale, Centre Mémoire de Ressources et de Recherche, Clermont-Ferrand, France; Erasmus MC Desiderius School, NETHERLANDS

## Abstract

**Background:**

Few studies have addressed health-related quality of life in community-dwelling individuals aged 80 years and above and very few self-assessment quality of life questionnaires have been formally validated in these populations. This study aimed to validate a French version of the LEIPAD, a self-administered questionnaire assessing the health-related quality of life of people aged 80 years and over.

**Method:**

A cross-sectional study of people aged 80 years and over living at home in France was conducted. All subjects recruited were sent a letter explaining the study and requesting their consent to take part. Those who accepted then received the questionnaires, including the LEIPAD, which assesses health-related quality of life in the subjects aged 65 years and above. We assessed its psychometric properties: data completeness, score distribution, floor and ceiling effects, internal consistency, item-total correlations, inter-scale correlations, reliability and convergent validity with the Medical Outcome Study Short-Form 36 (SF-36).

**Results:**

The results obtained from 184 older people (mean age of 83.9 years, standard deviation 3.3) showed very good acceptability (missing data between 1.1% and 11.4% for LEIPAD scales) Factor analysis of the instrument confirmed the multi-dimensional structure in seven independent scales similar to the original version. Good internal consistency (Cronbach’s alpha ranging from 0.68 to 0.87) and strong test-retest reliability of the LEIPAD scales (intraclass correlation coefficients ranging from 0.77 to 0.95) were found. Convergent validity with the SF-36 showed moderate to strong correlations, consistent with the hypotheses stated.

**Conclusions:**

The validation of this specific questionnaire will make it possible to investigate individually the health-related quality of life of French older people living at home and will enable French-speaking investigators to contribute to national and international research projects.

## Introduction

The world’s population is ageing in virtually every country, a trend that is even more pronounced in Europe. In 2016, the number of people aged 80 years and above was over 130 million; it is expected that this number will rise to 315 million by 2040. People aged 80 years and above will represent 3.5% of the world’s population [1] and 9.1% of the European population [2].

A number of health conditions exist that can substantially affect the health-related quality of life (HRQoL) of older population [3]. Hence the appropriate assessment of HRQoL in older people is assuming greater importance in research projects gauging the performance and economic evaluations of health systems.

When we started the cross-cultural evaluation of the French version of the LEIPAD, in 2007, few self-administered instruments had been developed to assess HRQoL specifically in older people. Some had good psychometric properties (LEIPAD, Perceived Well-being Scale, Quality of Life Profile—Seniors Version, Wellness Index, CASP-19 and the WHOQOL-Old, a complementary module of the WHOQOL-100 or the WHOQOL-BREF) [4]. To our knowledge at that time, these scales were not available and formally validated in French [4,5]. We were looking for a scale that would allow the assessment of older people’s HRQoL in a context of medical and psychosocial interventions. The LEIPAD, an acronym deriving from the first two of the three universities involved in its development (LEIden in the Netherlands and PADua in Italy) is a questionnaire especially designed in 1998 in English for self-assessment of HRQoL for community–dwelling subjects aged 65 years and above [6]. It was developed by the European office of WHO, with the aim of providing an instrument that could be easily used in clinical assessment, and that could be applied to different cultural settings. It was validated in an initial study of 586 people aged 72.5 years (standard deviation SD 5.9) [6], including quite a few adults aged 80 years and above. It showed good psychometric characteristics [4–6].

The LEIPAD was cross-culturally adapted in French for self-assessment by our team [5]. However, in our validation of the French version of the LEIPAD, only 32 participants were aged 80 years and above [5,7] which called into question the validity of the questionnaire in this age group. Patient-reported outcome measures, such as HRQoL, can now be used only after their psychometric characteristics have been validated for the population of interest. Thus, we decided to perform a new study to assess the psychometric properties of the French version of the LEIPAD questionnaire in a large sample of community-dwelling individuals aged 80 years and above.

Guidelines for assessing the validity of a HRQoL questionnaire have been defined and cover eight quality criteria [8]: content validity, floor and ceiling effects, internal consistency, criterion validity, construct validity or convergent validity, reliability, responsiveness, and interpretability. Content validity of the questionnaire was performed by De Leo *et al*. at the time of its initial development [6]. Criterion validity cannot be tested for the LEIPAD as there is no gold standard for the measurement of HRQoL in people aged 80 years and above. Responsiveness and interpretability were not tested in our cross-sectional study.

The psychometric properties evaluated in the present study, therefore, were acceptability (relevance), floor and ceiling effects, internal consistency, convergent validity and reliability.

## Methods

### Study design

The project was approved by the French regional ethics committee “Comité d’Ethique des Centres d’Investigation Clinique de l’Inter-région Rhône-Alpes-Auvergne—CE-CIC Grenoble” (IRB 00005921) and conducted according to the principles expressed in the Declaration of Helsinki. All subjects enrolled gave their written informed consent.

Adults aged 80 years and over who were not living in an institution were identified from the records of six town councils of a French territorial division. Each council gave a list containing the identities and the household addresses of older inhabitants, of whom 2071 were identified as eligible to participate.

The study design was the same as that previously described in detail for the validation in people aged 65 years and above [5]. Over a period of eight months, 1501 subjects were selected at random to participate. This sample size was calculated on the basis of two hypotheses: (i) the participation rate in the study would be lower in older people (10% rather than the overall rate of 15% in the previous study with the same design in subjects aged 65 years and over [5]); (ii) a subject-item ratio of 5 is required as an adequate size for factor analysis [8].

All the selected subjects were sent a letter explaining the aim of the study and asking if they would agree to participate. Adults who accepted were sent medical and socio-demographic questionnaires, a life events questionnaire, the French culturally adapted version of the LEIPAD, the SF-36 [9] and a letter explaining how to complete these questionnaires.

We randomly selected 110 subjects among respondents (the sample size required for an estimation of intraclass correlation coefficient of 0.50 with power of 80% and alpha of 5% and with an estimated response rate to test-retest of 20%) to study the reliability of the LEIPAD by test-retest [10]. The set of questionnaires was administered a second time about 15 days after the initial assessment [8]. Only respondents indicating no change in health status were retained in this analysis, as recommended [11]. Those who declared any additional health problems or treatment modifications since the first evaluation and who mentioned any events that would have disrupted their life between test and retest were excluded from reliability analysis.

### Participants

Patient inclusion criteria were: at least 80 years of age; living at home; not living in an institution; without dementia syndrome or other neurodegenerative diseases; and capable of completing the questionnaires without help.

Once all the data were collected, subjects who had not completed the questionnaires on their own (identified by a question to that effect) and who were suspected of having a neurodegenerative disease (identified by an item about their current treatment) were removed from the analysis.

### Instruments administered

The French versions of the LEIPAD [5] (S1 Fig) and of the SF-36 questionnaires [9,12] were self-administered.

#### LEIPAD

The LEIPAD questionnaire comprises 31 items [6] which are grouped into seven scales forming the core of the instrument: ‘Physical function’ (5 items), ‘Self-care’ (6 items), ‘Depression and anxiety’ (4 items), ‘Cognitive functioning’ (5 items), ‘Social functioning’ (3 items), ‘Sexual functioning’ (2 items), and ‘Life satisfaction’ (6 items). Each of these items is rated on a 4-point Likert scale, from 0 (corresponding to the best condition) to 3 (the worst condition).

For each scale a total score is calculated by adding the individual scores of the items making up the scale (only if all items are completed), with lower scores referring to good conditions.

#### SF-36

The SF36 is a general self-assessed instrument for measuring quality of life that is available in French. It is the most frequently used questionnaire for older people and has among the best metric properties [13]. The SF-36 consists of 36 items assigned to eight multi-item scales: ‘Physical functioning’, ‘Role physical’, ‘Mental health’, ‘Role emotional’, ‘Social functioning’, ‘Bodily pain’, ‘Vitality’, and ‘General health’. For each scale, the subject obtains a score between 0 and 100, with higher values indicating better HRQoL.

Demographic characteristics, current health problems (according to ICD-10 classification) and treatments, negative life events that may have disrupted their life during the last twelve months (such as bad health, hospitalisations, bereavements, financial worries, or conflicts with children) were also recorded.

### Statistical analyses

Statistical analyses were performed with SAS v9.4 software for Windows. P-values <0.05 were considered to be statistically significant.

#### Data completeness, score distribution, floor and ceiling effects

Data completeness (or respondent acceptability) of the LEIPAD was determined by looking at the frequency of missing values for each scale.

The distribution of the LEIPAD scores was evaluated using mean, standard deviation, range and median. For each scale of the LEIPAD floor effect (percentage of patients scoring at minimum level) and ceiling effect (percentage scoring at maximum level) were investigated. These effects were present if more than 15% of the subjects obtained the lowest or highest possible score [8].

#### Factor analysis

Factor analysis, with principal axis extraction method and oblique promax rotation (assuming that the factors are correlated), was performed to study the multidimensionality and distribution of the items in the hypothesized original scales. As the perception and definition of HRQoL varies from culture to culture, there was no guarantee that the French version reproduced the seven scales of the original instrument. We therefore performed an exploratory analysis to determine the underlying factor structure of the items [14–17]. Eigenvalues higher than one (Kaiser criterion) and Cattell’s scree plot [18] were used to verify factor solution accuracy. The solution giving the most adequate factor structure (item loadings greater than 0.32, no or few item cross loadings, i.e. no or few items with loadings at 0.32 or higher on two or more factors) was retained [19].

#### Internal consistency

Internal consistency was estimated by Cronbach’s α coefficient for multi-item scales [20]. The minimum required for the coefficient was 0.70, according to the standard used for group comparisons [21]. For the scale composed of two items, Spearman correlation coefficient was calculated.

#### Item-total correlations

Item-total correlations were used to evaluate the extent of the linear relationship between an item and its scale, corrected for overlap [22]. A minimum correlation coefficient of 0.40 was considered indicative of good item-total consistency [23].

#### Inter-scale correlations

Spearman’s coefficients were used to evaluate inter-scale correlations. Hypotheses regarding the relationships between the seven scales of the LEIPAD were made in terms of direction and magnitude, based on our experience. Correlations were considered very small for coefficients lower than 0.30, small for coefficients between 0.30 and 0.50, moderate from 0.50 to 0.70 and strong if higher than 0.70 [24]. Positive correlations were expected for all scales. The ‘Physical function’ scale was expected to correlate highly with the ‘Self-care’ scale (assessing physical tasks) and ‘Depression and anxiety’ scale (because items 6 and 7 related to sleep and tiredness could be symptoms of depression and anxiety). The ‘Self-care’ scale was expected to have small to moderate correlations with the ‘Depression and anxiety’ and ‘Cognitive functioning’ scales. The scales assessing mental health (‘Depression and anxiety’, ‘Cognitive functioning’, ‘Social functioning’ and ‘Life satisfaction’) are hypothesized to correlate moderately with one another. The ‘Sexual functioning’ scale was expected to have small correlations with the other scales.

#### Reliability

Reliability was explored by test-retest measures. Intraclass correlation coefficients (ICCs) [25], based on the two-way random effect model, were calculated for each of the LEIPAD scales. Coefficients higher than 0.70 were considered satisfactory [8].

#### Convergent validity

Convergent validity was obtained by studying the relationships between the LEIPAD and SF-36 scales and calculating Spearman’s correlation coefficients. Hypotheses about correlations were made in terms of magnitude, based on our experience. Negative correlations were expected between the LEIPAD and SF-36 scales because low scores indicated good conditions for the LEIPAD but bad conditions for the SF-36. Moderate to high correlations were expected between the LEIPAD ‘Physical function’ scale and the SF-36 ‘Physical functioning’, ‘Role physical’, ‘Bodily Pain’, ‘Vitality’ and ‘General health’ scales, and between the LEIPAD ‘Self-care’ scale and the SF-36 ‘Physical functioning’ scale. The LEIPAD ‘Depression and anxiety’ scale was expected to be correlated with the SF-36 ‘Mental health’, ‘Role Emotional’, and ‘Vitality’ scales. The LEIPAD ‘Cognitive functioning’ scale was expected to correlate with the mental dimensions of SF-36. The LEIPAD ‘Social functioning’ scale was hypothesized to be poorly correlated with the SF-36 ‘Social functioning’ scale, as the items making up these dimensions differed greatly between the two instruments. The LEIPAD ‘Sexual functioning’ scale was expected to correlate with the SF-36 ‘Physical functioning’, and the LEIPAD ‘Life satisfaction’ scale with the SF-36 ‘General health’ scale.

## Results

### Description of the participants

**Fig 1:**
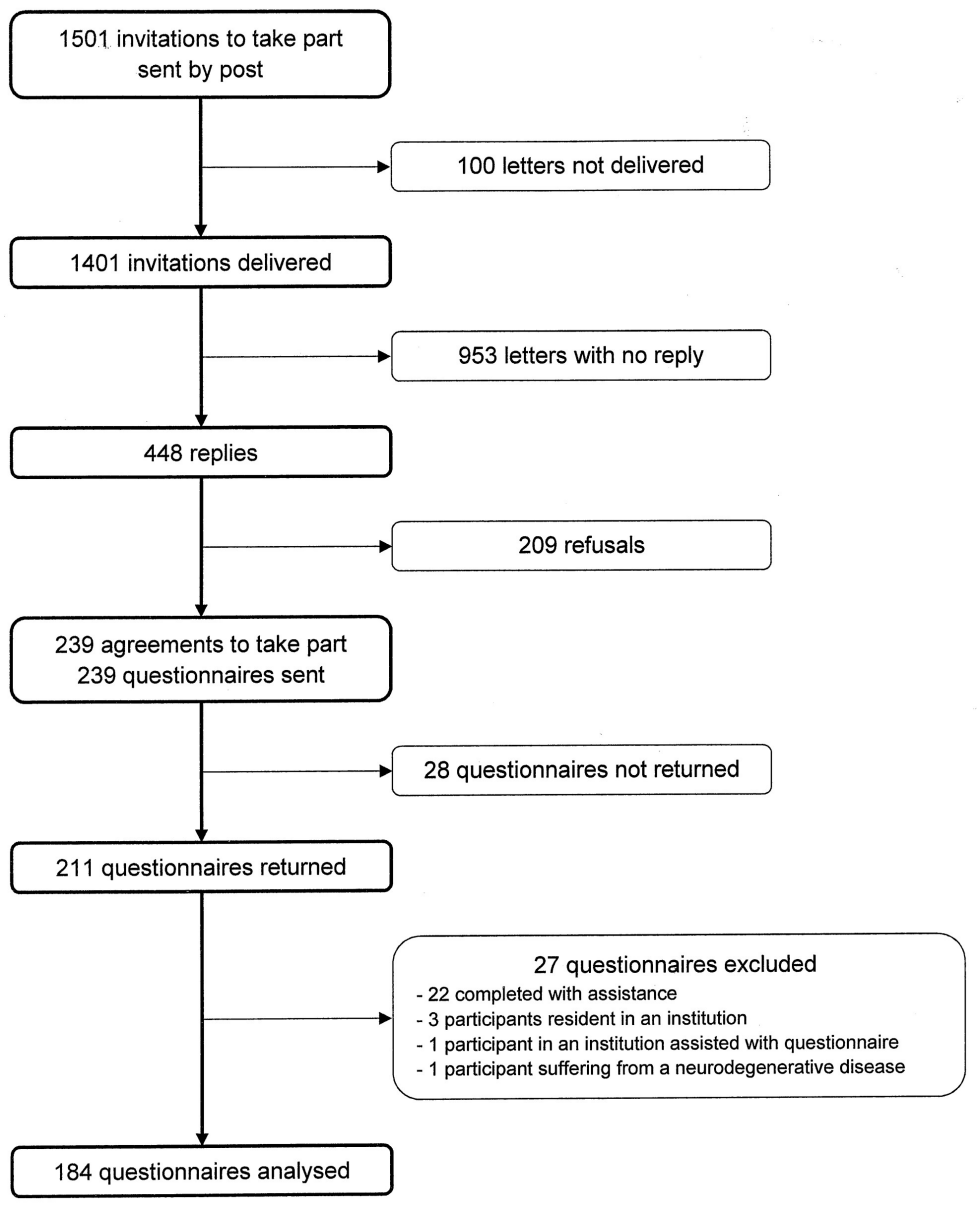
Organization chart of participation in the study.

Of the 1501 letter sent, 100 were not able to be distributed. Overall 448 subjects (32.0%) responded (Fig 1) and of these 239 (53.3%) agreed to enrol in the study. A comparison of subjects who agreed to participate and those who declined showed that the former were a little younger (85 years (SD 3.6) vs. 85.8 years (SD 3.8), p = 0.0349) and had a higher level of education (p<0.0001). No differences in terms of sex and location existed (p = 0.1442 and p = 0.5001 respectively).

Of the 239 older adults who agreed to participate, 211 (88.3%) returned the questionnaires. Twenty seven adults were then excluded because they did not fulfil the inclusion criteria. (Fig 1). Thus analyses were made of the data of 184 participants, who were aged between 80 and 95 years, with a mean age of 83.9 years (SD 16.3).

The sociodemographic characteristics of the respondents are shown in Table 1.

**Table 1:** Respondents’ characteristics (n = 184).

	n	%
Age class		
80–84 years	113	61.4
85–95 years	71	38.6
Sex		
Men	83	45.1
Women	101	54.9
Marital status		
Never married	5	2.8
Married or living in a couple	106	58.6
Widowed	66	36.5
Divorced	4	2.2
Education		
Lower than high school	143	78.6
Equal to high school or higher	39	21.4
Hospitalisation in the last year		
Yes	41	23.0
No	137	77.0
Driving a car		
Yes	109	61.6
No	68	38.4
Number of health problems ^a^	4.4 (2.0)	
None	4	2.2
At least one	180	97.8
ICD-10 classification of health problems		
Diseases of the circulatory system	147	79.9
Diseases of the musculoskeletal system and connective tissue	118	64.1
Endocrine, nutritional and metabolic diseases	117	63.6
Diseases of the eye and adnexa	89	48.4
Diseases of the ear and mastoid process	84	45.7
Diseases of the digestive system	64	34.8
Mental and behavioural disorders	53	28.8
Diseases of the genitourinary system	47	25.5
Diseases of the nervous system	37	20.1
Diseases of the respiratory system	35	19.0
Diseases of the blood and blood-forming organs and certain disorders involving the immune mechanism	14	7.6
Neoplasms	5	2.7
Certain infectious and parasitic diseases	4	2.2
Diseases of the skin and subcutaneous tissue	1	0.5

^a^ Mean (SD)

### Data completeness, score distribution, floor and ceiling effects

The descriptive statistics and score distributions for the LEIPAD scales are given in Table 2. The percentage of missing values per LEIPAD scale was low, with values ranging from 1.1% for the ‘Social functioning’ scale to 11.4% for the ‘Life satisfaction’ scale. Even the scale related to sexuality had a very low percentage of missing values (4.3%).

**Table 2:** Descriptive statistics and score distributions of the LEIPAD scales (n = 184).

LEIPAD scales *(/highest possible score)*	Missing values *(%)*	Mean (SD)	Range	Median	Floor effect (%)	Ceiling effect *(%)*
Physical function *(/15)*	3.8	5.3 (2.6)	0–13	5	1.1	0
Self-care *(/18)*	6.0	2.1 (3.1)	0–14	0	50.9	0
Depression and anxiety *(/12)*	2.7	1.8 (2.2)	0–12	1	40.8	0.6
Cognitive functioning *(/15)*	6.0	3.3 (2.5)	0–11	3	3.5	0
Social functioning *(/9)*	1.1	2.1 (1.7)	0–9	2	18.7	0.5
Sexual functioning *(/6)*	4.3	5.2 (1.1)	2–6	6	0	59.1
Life satisfaction *(/18)*	11.4	6.1 *(2*.*5)*	0–14	6	0.6	0

SD: Standard Deviation

Floor effects were obtained for the ‘Self-care’, ‘Depression and anxiety’ and ‘Social functioning’ scales. No ceiling effect was found except for the ‘Sexual functioning’ scale.

### Factor analysis

The 31 item structure of the LEIPAD was tested by factor analysis with promax rotation. Five factors accounting for 87.4% of the total variance were extracted and gave the best factor structure solution (Table 3). Most of the items loaded higher on their hypothesized scale than on other scales.

**Table 3:** Factor loadings from the factor analysis on the LEIPAD questionnaire.

Items	Factor 1	Factor 2	Factor 3	Factor 4	Factor 5
Self-care					
Item 2	**0.80**	-0.04	0.02	-0.11	0.05
Item 3	**0.74**	-0.02	-0.03	0.04	-0.13
Item 4	0.18	-0.20	0.00	0.11	0.13
Item 5	**0.84**	-0.06	-0.03	0.12	-0.18
Item 10	**0.89**	-0.04	-0.10	0.00	-0.01
Item 11	**0.90**	-0.02	-0.08	-0.15	0.03
Physical function					
Item 1	**0.34**	0.19	0.17	0.16	0.13
Item 6	0.26	0.04	0.12	0.01	**0.37**
Item 7	**0.34**	0.13	0.16	-0.01	0.29
Item 9	**0.62**	0.10	-0.05	0.00	0.23
Item 12	**0.68**	0.04	0.11	0.09	0.01
Cognitive functioning					
Item 8	-0.03	**0.56**	-0.08	0.08	0.27
Item 13	0.03	**0.72**	-0.08	-0.28	0.05
Item 14	-0.09	**0.82**	0.14	-0.07	-0.02
Item 15	-0.11	**0.70**	-0.14	0.17	-0.02
Item 16	-0.10	**0.87**	0.01	0.03	-0.07
Depression and anxiety					
Item 17	0.02	**0.68**	0.01	0.04	0.18
Item 18	0.08	**0.81**	0.01	0.01	-0.04
Item 19	0.12	**0.44**	0.24	0.20	-0.10
Item 20	0.17	**0.64**	0.21	0.02	-0.22
Social functioning					
Item 21	-0.04	-0.04	**0.74**	-0.06	0.09
Item 22	-0.15	0.05	**0.80**	0.10	0.03
Item 23	0.14	0.14	**0.38**	-0.18	0.10
Life satisfaction					
Item 26	0.14	0.07	**0.57**	0.02	0.03
Item 27	-0.03	0.07	-0.18	**0.70**	0.13
Item 28	0.03	-0.04	0.04	**0.69**	0.15
Item 29	0.07	-0.16	**0.52**	**0.34**	-0.03
Item 30	-0.14	-0.01	**0.40**	**0.34**	-0.14
Item 31	0.15	0.18	0.19	**0.35**	-0.03
Sexual functioning					
Item 24	-0.02	-0.04	0.00	0.07	**0.65**
Item 25	-0.04	0.03	0.03	0.13	**0.59**

Loadings higher than 0.32 are presented in bold.

The items of the ‘Physical function’ and ‘Self-care’ scales loaded higher than 0.32 on the same factor 1, with two exceptions: item 4 (being able to eat without help) and item 6 of ‘Physical function’ scale (sleep problems), which loaded on factor 5 (‘Sexual functioning’ scale). The items of ‘Cognitive functioning’ and ‘Depression and anxiety’ scales loaded higher than 0.32 on the same factor 2. Those of the ‘Social functioning’ and ‘Sexual functioning’ scales loaded respectively on factors 3 and 5. The ‘Life satisfaction’ scale was split into factors 3 and 4. Items 29 and 30 loaded higher than 0.32 both on both factors 3 and 4. Items 26, 29 and 30 had loadings greater than 0.32 on factor 3, the axis of the ‘Social functioning’ scale. These items were related to hobbies, satisfaction with life at present when compared to the past and expectations for the future.

### Internal consistency

The LEIPAD scales showed good internal consistency with Cronbach’s α ranging from 0.68 to 0.87 (Table 4). The ‘Social functioning’ and ‘Sexual functioning’ scales did not obtain the minimum required coefficient of 0.70. However Cronbach’ α coefficients were 0.69 and 0.68 respectively, values very close to 0.70.

**Table 4:** Internal scale consistency (Cronbach’s α) and inter-scale correlations for LEIPAD.

LEIPAD scales	Physical function	Self-care	Depression and anxiety	Cognitive functioning	Social functioning	Sexual functioning	Life satisfaction
Physical function	**0.81**						
Self-care	0.71*	**0.82**					
Depression and anxiety	0.58*	0.37*	**0.87**				
Cognitive functioning	0.47*	0.29*	0.64*	**0.85**			
Social functioning	0.41*	0.30*	0.46*	0.41*	**0.69**		
Sexual functioning	0.30*	0.22*	0.22*	0.14	0.08	**0.68**	
Life satisfaction	0.43*	0.28*	0.43*	0.36*	0.37*	0.10	**0.72**

Cronbach’s α for multi-item scales are reported on the diagonal and in bold text (Spearman correlation coefficients were calculated for two-item scales).

Inter-scale correlations are Spearman coefficients.

* Significantly different from 0 (p<0.05).

### Item-total correlations

Corrected item-total correlations ranged from 0.37 to 0.79 and they were higher than the 0.40 required, except for three items, which had values of 0.37, 0.38 and 0.39, all very close to 0.40. These correlations indicate good item-total consistency.

### Inter-scale correlations

Hypothesized positive relationships were found between the seven LEIPAD scales (Table 4). The highest correlation was obtained between the ‘Physical function’ and ‘Self-care’ scales (r = 0.71). Moderate correlations were observed between the ‘Physical function’ and ‘Depression and anxiety’ scales (r = 0.58) and between the ‘Self-care’ and ‘Depression and anxiety’ scales (r = 0.37). An almost moderate correlation was found between the ‘Self-care’ and ‘Cognitive functioning’ scales (r = 0.29). Scales assessing mental health (‘Depression and anxiety’, ‘Cognitive functioning’, ‘Social functioning’ and ‘Life satisfaction’) were moderately correlated with one another, with correlation coefficients ranging from 0.36 to 0.64. The ‘Sexual functioning’ scale had small correlations with the other scales (ranging from 0.08 to 0.22), except for the ‘Physical function’ scale with a moderate correlation of 0.30. All but three inter-scale correlations (between the ‘Sexual Functioning’ scale and the ‘Cognitive functioning’ (r = 0.14), ‘Social functioning’ (r = 0.08) and ‘Life satisfaction’ (r = 0.10) scales) were significant (p<0.05) with a range from 0.22 to 0.71.

### Reliability

Of the participants selected for the retest, 87 (79.1%) returned completed questionnaires. We checked for inclusion criteria and that the respondents had maintained stable health status between the two evaluations. Thirty four subjects were excluded from the reliability analysis, 7 because of exclusion criteria (5 did not fill the questionnaires alone, 1 lived in an institution and 1 was suspected of having a neurodegenerative disease) and 27 because of events that had disrupted their life between test and retest. No patient declared any additional health problems or treatment modifications since the first evaluation. Finally, 53 respondents were retained. All ICCs for LEIPAD scales were greater than 0.70 (Table 5) ranging from 0.77 to 0.95.

**Table 5:** Test-retest reliability for the LEIPAD scales (n = 53).

LEIPAD scales	ICC [95% CI]
Physical function	0.93 [0.88–0.97]
Self-care	0.95 [0.91–0.98]
Depression and anxiety	0.89 [0.80–0.94]
Cognitive functioning	0.77 [0.62–0.87]
Social functioning	0.84 [0.73–0.91]
Sexual functioning ^(^*^)^	0.91 [0.85–0.95]
Life satisfaction	0.78 [0.63–0.87]

ICC: Intraclass Correlation Coefficient

95% CI: 95% Confidence Intervals

^(^*^)^ Spearman correlation coefficients.

## Convergent validity

Correlations between the LEIPAD and SF-36 scales were negative (Table 6). All LEIPAD scales, except the ‘Sexual functioning’ scales, were significantly correlated with all the scales of the SF-36 (p<0.05). The LEIPAD ‘Physical function’ scale was highly correlated with the SF-36 ‘Physical functioning’ (r = -0.75), ‘Role physical’ (r = -0.70) and ‘Vitality’ (r = -0.70) scales, and moderately correlated with the other SF-36 scales (correlation coefficients ranging from -0.49 to -0.69). Correlation between the LEIPAD ‘Self-care’ scale and the SF-36 ‘Physical functioning’ scale was high (r = -0.78). The LEIPAD ‘Depression and anxiety’ scale was strongly correlated with the SF-36 ‘Mental health’ scale (r = -0.78), and moderately correlated with the SF-36 ‘Role emotional’ (r = -0.55) and ‘Vitality’ (r = -0.52) scales. The LEIPAD ‘Cognitive functioning’ scale correlations with the SF-36 scales were mostly small (r = -0.25 to -0.49), with the sole exception of the SF-36 ‘Mental health’ scale, which was moderate (r = -0.57). The LEIPAD and SF-36 ‘Social functioning’ scales were rather moderately correlated (r = -0.49). The LEIPAD ‘Sexual functioning’ scale was poorly correlated with the SF-36 scales (r = -0.10 to -0.32), even with the ‘Physical functioning’ scale (r = -0.28). Correlations between the LEIPAD ‘Life satisfaction’ scale and the SF-36 scales were small (r = -0.26 to –0.48), with ‘Bodily pain’ (r = -0.48) and ‘General health’ (r = -0.47) scales being rather moderately correlated.

**Table 6:** Spearman’s correlations between the LEIPAD and the SF-36 scales.

	*SF-36 scales*							
*LEIPAD scales*	Physical functioning	Role physical	Mental health	Role emotional	Social functioning	Bodily pain	Vitality	General health
Physical function	-0.75*	-0.70*	-0.58*	-0.49*	-0.51*	-0.67*	-0.70*	-0.69*
Self-care	-0.78*	-0.53*	-0.38*	-0.36*	-0.44*	-0.51*	-0.56*	-0.47*
Depression and anxiety	-0.35*	-0.41*	-0.78*	-0.55*	-0.45*	-0.38*	-0.52*	-0.48*
Cognitive functioning	-0.26*	-0.31*	-0.57*	-0.39*	-0.34*	-0.25*	-0.49*	-0.35*
Social functioning	-0.26*	-0.20*	-0.44*	-0.30*	-0.49*	-0.33*	-0.37*	-0.34*
Sexual functioning	-0.28*	-0.24*	-0.27*	-0.15	-0.09	-0.18*	-0.32*	-0.10
Life satisfaction	-0.34*	-0.31*	-0.47*	-0.26*	-0.45*	-0.48*	-0.43*	-0.47*

Moderate and high correlations are presented in bold (|r|> 0.50).

* Significantly different from 0 (p<0.05).

SF-36: Medical Outcome Study Short-Form 36

## Discussion

This study presents for the first time a psychometric analysis of the culturally adapted French version of the LEIPAD questionnaire in a population aged 80 years old and over living at home.

In our first cross-cultural evaluation of the French LEIPAD [5], the number of adults enrolled above the age of 80 was small, and so valid results were obtained only for the subsample of those younger than 80 years old. The proportion of the world’s population of people over 80 is going to increase drastically. Health problems and dependency increase with aging and so we thought it would be valuable to enrich the psychometric evaluation of the LEIPAD in a similar study specifically concerning this age group. The survey we undertook benefited from several important methodological factors. Our sample size is to our knowledge, after a reference database search, one of the largest to be used in a study of the psychometric properties of self-assessed instruments of HRQoL in community-dwelling people aged 80 years and above [4]. Our study population was comparable to those in other published reports with regard to sociodemographic characteristics (French sample of 168 people in the ESEMeD study [26]), living alone or not [27,28], driving a car [29,30] and medical characteristics [28] except for the rate of eye diseases. According to the ICD 10 classification it was lower than that recorded in the general population of the same age [31] possibly because subjects unable to complete the questionnaires without help were not included in our study.

While older age groups often have higher frequencies of missing data [32], the French version appeared to have very good acceptability in people aged 80 years and above with low percentages of missing values, even for the scale related to sexuality. This last result is rarely mentioned. Indeed only a few studies have been made on the sexuality of older adults (up to the age of 78 years) in the general population [33–35]. Some studies of HRQoL in people up to the age of 80 years have included this dimension but only one, in which there were only 8.7% missing data for items related to sexual activity, has addressed the topic specifically in older people [36]. We found a ceiling effect solely for the ‘Sexual functioning’ scale, which shows that the only major health problem influencing their HRQoL was sexuality. That sexuality influences HRQoL in people aged 80 years and above is not surprising, and yet the literature on this topic is scarce [37,38]. It could be that problems with sexual functioning were the only major problem in our participants who where living at home and relatively self-sufficient. At the same time, it could be that the ‘Sexual functioning’ scale is too weak to pick up variances in the concept of sexual functionality. The high floor effect of the ‘Self-care’ scale was certainly due to the fact that respondents were home-dwelling and were relatively self-sufficient (for example, 100% were able to eat without help—item 4). There was also a floor effect for the ‘Depression and anxiety’ scale, which can be explained in part by the state of health and personal autonomy of the participants, and for the ‘Social functioning’ scale, probably owing to the same mechanisms. This is consistent with similar levels of autonomy observed in other surveys [28,39] and with results reported in the dimension of ‘Anxiety and Depression’ in the EQ-5D [26,40]. These observed floor and ceiling effects are not due to any flaw in the LEIPAD questionnaire but rather to the relatively healthy status of our study subjects, who were still living at home and relatively self-sufficient.

The results of our study supported construct validity of the LEIPAD instrument in people aged 80 years and above, with a multidimensional structure in five independent scales. Item 4 had no loadings greater than 0.32 on factors, because of all subjects gave the same reply. The fact that item 6 (sleep problems) of the ‘Physical function’ scale was related to sexuality could reflect that the changes in sleep and sexuality that occur in old age. Whatever the case, it does not really affect the construct validity of the ‘Physical function’ scale. The relation of ‘Cognitive functioning’ to ‘Depression and anxiety’ is consistent with the well-known impact of depression/anxiety on cognitive functioning, particularly in old age [41]. Likewise, the relation of ‘Self-care’ to ‘Physical function’ is consistent with the documented effects of physical condition on the ability to take care of oneself in old age [42]. The fact that the ‘Life satisfaction’ scale is split into two close factors, illustrates that the concept itself of life satisfaction is somewhat heterogeneous. However, it is still worth keeping this dimension as a single entity because of its Cronbach’s alpha coefficient greater than the required value of 0.70.

The internal consistency of the scales was good for four scales (Cronbach’s α greater than 0.80) and acceptable for three scales (Cronbach’s α of 0.68, 0.69 and 0.72). Test-retest reliability, which is an essential property [43], was good since strong ICCs were obtained for all scales. Convergent validity was established by relating LEIPAD scores to the generic SF-36 scores, with the expected correlations found.

The present study provides evidence of the good psychometric properties of the French version of the LEIPAD in a community-dwelling population aged 80 years and over but has certain limitations. First, of the subjects contacted to take part in our study, only 12.3% were finally involved in the analysis, a proportion close to that in other surveys of this type among older people with postal administration [5,44]. Those who agreed to participate were a little younger and had a higher level of education than those who declined. This is not a major limit to representativeness as our purpose was not to assess the HRQoL in the older people but to study the psychometric properties of the French version of the LEIPAD. Second, we did not assess the cognitive status of the subjects before they replied to the questionnaire. However, we discarded the questionnaires of those receiving treatment generally prescribed for cognitive disorders and those who did not complete the questionnaire unaided.

## Conclusion

In conclusion, our study provides evidence of the good psychometric properties of the French version of the LEIPAD self-report questionnaire in people aged 80 years and above, similar to those in subjects younger than 80. Future work, on a larger representative sample in cross-sectional studies, should make it possible to establish population norms in older adults. Responsiveness (the ability of the LEIPAD instrument to detect changes in HRQoL over time) and interpretability (minimal important change, smallest detectable change, response shift) were not studied. Future longitudinal surveys assessing the questionnaire’s sensitivity to change need to be performed. Our study sample was composed of adults without any major health problems that would make them unable to continue living at home. It would be useful to evaluate the LEIPAD questionnaire among older people with significant somatic diseases [45–48] or psychiatric disorders [49,50] barring cognitive disorders, which are incompatible with the self-completion of questionnaires.

This cross-cultural adaptation of the LEIPAD for people aged 80 years and above is an extension of our previous work and will allow investigators to propose all French-speaking elderly subjects living at home as participants in national and international collaboration research projects using this scale.

## Supporting information

S1 Fig French version of LEIPAD questionnaire. (DOCX)
