# Supplementary material for: Validation of the French version of the LEIPAD in community-dwelling people aged 80 years and above
Source: PLoS One. 2019 Mar 19;14(3):e0213907. doi: 10.1371/journal.pone.0213907 (PMC6424406; doi:10.1371/journal.pone.0213907)
Supplement: S1 Fig — (DOCX) [file pone.0213907.s001.docx]

**S1 Fig : French version of LEIPAD questionnaire**

**Questionnaire LEIPAD version française**

**INSTRUCTIONS**

Ce questionnaire traite des aspects courants de votre **vie quotidienne actuellement.**

Merci de lire chaque question attentivement et de cocher la réponse la plus appropriée à votre situation.

Par exemple, si on considère les questions suivantes:

**"Etes-vous capable de marcher sans aide?"**

Quand vous êtes capable de marcher sans aide, mais avec difficulté, alors cochez comme suit:

**□** Pas capable du tout

**□** Seulement avec l’aide de quelqu’un

**□** Avec difficulté mais sans aide

**□** Sans difficulté

**Date de naissance**

Quand vous êtes né(e) le 17 Juillet 1922, les cases à côté de la date de naissance doivent être remplies comme suit:

jour l***1*** l***7*** l mois l***0*** l***7*** l année l***1*** l***9*** l***2*** l***2*** l

Mode de cohabitation

Dans le cas où vous vivez dans votre propre maison avec votre conjoint(e), alors la question sur le mode d’habitation doit être cochée de la façon suivante :

**□** Seul(e)

**□** Avec d’autres personnes avec lesquelles vous avez des liens

**□** Avec d’autres personnes dans les mêmes lieux mais sans autre lien

**□** Dans une institution

Attention!

Concernant les questions à choix multiples, vous ne devez cocher qu’une seule réponse! Si vous avez un doute, merci de choisir la réponse la plus proche de votre situation actuelle.

**Nous vous remercions de remplir seul(e) ce questionnaire et de ne pas sauter de questions!**

**Merci de compléter cette page en premier.**

**Date de naissance:** jour l_ l_ l mois l_ l_ l année l_ l_ l_ l_ l

Sexe:

□ Masculin

□ Féminin

Statut marital:

□ Jamais marié(e)

□ Marié(e) ou vivant en couple

□ Veuf (veuve)

□ Divorcé(e)

**Niveau d’études:**

□ Ecole Primaire □ Première partie de BAC

□ Certificat d’Etude □ BAC

□ Examen d’entrée en 6^ème^  □ Diplôme autre que BAC ou Reconnaissance professionnelle

□ BEPC/Brevet □ Etudes Supérieures

□ CAP □ Diplômes obtenus pendant la vie professionnelle (après le BAC ou niveau BAC)

**Nombre d’années d’études:** .l__l__l

(Totalisez le nombre d’années d’études depuis l’entrée en Ecole Primaire)

Occupation:

□ Activité(s) rémunérée(s)

(profession actuelle …………………………………………………)

□ Retraité(e) et /ou travailleur(se) bénévole

(dernière profession ………………………………………………...)

□ Femme au foyer

Mode de cohabitation:

□ Seul(e)

□ Avec d’autres personnes avec lesquelles vous avez des liens

□ Avec d’autres personnes dans les mêmes lieux mais sans autre lien

□ Dans une institution

Continuez maintenant avec la première question de la page suivante et ainsi jusqu’à la fin du questionnaire.

**Toutes ces questions font référence à votre situation actuelle.**

1. Comment qualifieriez-vous votre état de santé global?

**□** Excellent

**□** Bon

**□** Pas très bon

**□** Mauvais

2. Etes-vous capable de monter et de descendre les escaliers sans aide?

**□** Sans difficulté

**□** Avec difficulté

**□** Seulement avec l’aide de quelqu’un

**□** Pas capable du tout

3. Etes-vous capable de vous habiller entièrement seul(e)?

**□** Sans difficulté

**□** Avec difficulté

**□** Seulement avec l’aide de quelqu’un

**□** Pas capable du tout

4. Etes-vous capable de manger sans aide?

**□** Sans difficulté

**□** Avec difficulté

**□** Seulement avec l’aide de quelqu’un

**□** Pas capable du tout

**Toutes ces questions font référence à votre situation actuelle.**

5. Etes-vous capable de prendre un bain ou une douche sans aide?

**□** Sans difficulté

**□** Avec difficulté

**□** Seulement avec l’aide de quelqu’un

**□** Pas capable du tout

6. Avez-vous des problèmes de sommeil?

**□** Non, pas du tout

**□** Oui, mineurs

**□** Oui, modérés

**□** Oui, très sévères

7. Vous sentez-vous fatigué(e), sans énergie?

**□** Jamais

**□** Quelquefois

**□** Assez souvent

**□** Très souvent

8. Avez-vous des difficultés à vous concentrer?

**□** Jamais

**□** Quelquefois

**□** Assez souvent

**□** Très souvent

**Toutes ces questions font référence à votre situation actuelle.**

9. Etes-vous capable d'effectuer vos tâches quotidiennes que ce soit chez vous, au travail, ou ailleurs?

**□** Oui, complètement

**□** En grande partie

**□** Seulement quelques unes

**□** Non, aucune

10. Pouvez-vous faire vos courses seul(e)?

**□** Sans difficulté

**□** Avec difficulté

**□** Seulement avec l’aide de quelqu’un

**□** Pas capable du tout

11. Pouvez-vous prendre les transports en commun?

**□** Sans difficulté

**□** Avec difficulté

**□** Seulement avec l’aide de quelqu’un

**□** Pas capable du tout

12. Vos problèmes de santé, si vous en avez, vous empêchent-ils de faire ce que vous voulez?

**□** Pas du tout

**□** Un peu

**□** Assez

**□** Beaucoup

**Toutes ces questions font référence à votre situation actuelle.**

13. Vous arrive-t-il de ne pas pouvoir penser clairement ou d'être embrouillé(e)?

**□** Très rarement

**□** Rarement

**□** Souvent

**□** Très souvent

14. Vos difficultés pour réfléchir, si vous en avez, vous empêchent-elles de faire ce que vous voulez?

**□** Pas du tout

**□** Un peu

**□** Assez

**□** Beaucoup

15. Avez-vous une bonne mémoire?

**□** Excellente

**□** Bonne

**□** Pas très bonne

**□** Mauvaise

16. Vos problèmes de mémoire, si vous en avez, vous empêchent-ils de faire ce que vous voulez?

**□** Pas du tout

**□** Un peu

**□** Assez

**□** Beaucoup

**Toutes ces questions font référence à votre situation actuelle.**

17. Globalement, vous sentez-vous anxieux(se)?

**□** Pas du tout anxieux(se)

**□** Un peu anxieux(se)

**□** Anxieux(se)

**□** Très anxieux(se)

18. Votre anxiété, si vous êtes anxieux(se), vous empêche-t-elle de faire ce que vous voulez?

**□** Pas du tout

**□** Un peu

**□** Assez

**□** Beaucoup

19. Globalement, vous sentez-vous déprimé(e)?

**□** Pas du tout déprimé(e)

**□** Un peu déprimé(e)

**□** Déprimé(e)

**□** Très déprimé(e)

20. Cette dépression, si vous êtes déprimé(e), vous empêche-t-elle de faire ce que vous voulez?

**□** Pas du tout

**□** Un peu

**□** Assez

**□** Beaucoup

**Toutes ces questions font référence à votre situation actuelle.**

21. Etes-vous satisfait(e) de vos contacts sociaux ou de vos relations avec les autres?

**□** Très satisfait(e)

**□** Satisfait(e)

**□** Insatisfait(e)

**□** Très insatisfait(e)

22. Vous-sentez vous heureux(se) dans vos relations avec les autres personnes?

**□** Beaucoup

**□** Assez

**□** Un peu

**□** Pas du tout

23. Avez-vous quelqu'un à qui parler de problèmes personnels quand vous le souhaitez?

**□** Presque toujours

**□** Assez souvent

**□** Quelquefois

**□** Pas du tout

24. Etes-vous intéressé(e) par la sexualité?

**□** Beaucoup

**□** Assez

**□** Un peu

**□** Pas du tout

**Toutes ces questions font référence à votre situation actuelle.**

25. Avez-vous des relations sexuelles?

**□** Souvent

**□** Assez souvent

**□** Quelquefois

**□** Pas du tout

26. Etes-vous satisfait(e) de la façon dont vous parvenez à organiser vos passe-temps ou vos loisirs?

**□** Très satisfait(e)

**□** Satisfait(e)

**□** Insatisfait(e)

**□** Très insatisfait(e)

27. Etes-vous satisfait(e) de votre situation financière?

**□** Très satisfait(e)

**□** Satisfait(e)

**□** Insatisfait(e)

**□** Très insatisfait(e)

28. Avez-vous l’impression de ne pas pouvoir vous permettre le niveau de vie dont vous auriez envie?

**□** Pas du tout

**□** Un peu

**□** Beaucoup

**□** Vraiment beaucoup

**Toutes ces questions font référence à votre situation actuelle.**

29. Etes-vous satisfait(e) de votre vie actuelle, si vous la comparez au passé?

**□** Très satisfait(e)

**□** Satisfait(e)

**□** Insatisfait(e)

**□** Très insatisfait(e)

30. Tout compte fait, comment voyez-vous les choses pour l'avenir?

**□** Bien mieux

**□** Mieux

**□** Pire

**□** Bien pire

31. Votre idée de l’avenir vous empêche-elle de faire ou d'entreprendre ce que vous voulez ?

**□** Pas du tout

**□** Un peu

**□** Pas mal

**□** Beaucoup

**IMPORTANT!**

C’était la dernière question. Merci de vérifier une nouvelle fois si vous n’avez pas oublié de répondre à une question.

**COMMENTAIRES**

Si vous avez des commentaires, positifs ou négatifs, sur ce questionnaire, merci de les écrire ci-dessous.

______________________________________________________________________________________________________________________________________________________

____________________________________________________________________________________________________

____________________________________________________________________________________________________

____________________________________________________________________________________________________

**Vous avez rempli ce questionnaire:**

**□** Entièrement seul(e)

**□** Avec l’aide de quelqu’un

**Nous vous remercions beaucoup pour votre participation.**
